# The ball kicking speed: A new, efficient performance indicator in youth soccer

**DOI:** 10.1371/journal.pone.0217101

**Published:** 2019-05-17

**Authors:** Ante Rađa, Goran Kuvačić, Andrea De Giorgio, Maha Sellami, Luca Paolo Ardigò, Nicola Luigi Bragazzi, Johnny Padulo

**Affiliations:** 1 Faculty of Kinesiology, University of Split, Split, Croatia; 2 Sport Performance Lab, University of Split, Split, Croatia; 3 Department of Psychology, University eCampus, Novedrate, Italy; 4 Sport Science Program (SSP), College of Arts and Sciences (CAS), Qatar University, Doha, Qatar; 5 School of Exercise and Sport Science, Department of Neurosciences, Biomedicine and Movement Sciences, University of Verona, Verona, Italy; 6 School of Public Health, Department of Health Sciences (DISSAL), University of Genoa, Genoa, Italy; 7 Tunisian Research Laboratory Sports Performance Optimization, National Center of Medicine and Science in Sport, Tunis, Tunisia; James Cook University College of Healthcare Sciences, BRAZIL

## Abstract

Success in different soccer skills like kicking depends on motor abilities achieved. Kicking is a soccer fundamental, which depends on many different and complex factors (technique, foot-ball interaction, ball flight, etc.). Therefore, it is important to identify players that are able to perform faster kicks using both dominant and non-dominant leg. The current study investigated some basic variables of different soccer kicking speed and their relevance to success in youth soccer academy. 119 players from the first and the second division participated to this study. They were randomly divided into age groups (U-15, U-17, and U19) and team *status* (first team, reserves). The diagnostic ability of the different ball kicking speed tests in capturing differences between first team players and reserves among different age categories were computed using the receiver operating characteristics analysis. Results demonstrated that first team players achieved better results when comparing to reserves in each category. In addition, differences were greater in the U-15 and the U-17 than in the U-19 age group. In conclusion, ball kicking speed could be one of the possible identification tools to evaluate players' success in youth soccer.

## Introduction

Soccer is a complex sport activity, whose success depends on various variables and factors, including physiological abilities and technical skills [1] and, among them, one of the most important is the kicking [2]. Soccer techniques improvement has recently been highlighted in the literature through an innovative training methodology for the young player [3]. Soccer includes different explosive movements like kicking and passing the ball, tackling, falling, jumping, starting, and stopping [4].

Lago-Peñas et al. objectified that players who shoot more during the match are more likely to be successful than others who shoot less [5]. This makes the development of proper shooting mechanics critically important and improving the kicking action is extremely crucial during the training of athletes. Kicking skills, however, are not easy tasks to achieve. In order to be correctly performed, they require a great degree of technical skills [6]. It should be noted that most kicks are usually done using feet (instep kick or side foot kick) [7,8].

The analysis of the dynamic movement of kicking has shown that side foot kick is more precise, whereas instep kick is the fastest type of kick in soccer [9,10]. To increase goal-scoring odds, player should reach the highest ball speed possible, which depends on several variables, such as the speed of the foot (distal segment) upon impact as well as the quality of the ball kick–foot impact [11–14]. In addition, it is desirable to have good kicking technique with both feet (dominant and non-dominant [15]). Furthermore, if the kick is faster it is less likely that the opposing goalkeeper or player will have enough time to react [16–18].

Rodríguez Lorenzo et al. [8] reviewed existing literature about the effects on *maximum* ball kicking speed of age, gender, limb dominance, practice duration, competition level, playing position, and variations in the kicking technique. At 15–19 years, kicking pattern is completely achieved (with *maximum* ball kicking speed = 80–103 km/h) [2]. Ball speed results significantly faster after a kick with the dominant leg compared with the non-dominant one from young subjects (86 *vs*. 74 km/h), through amateurs (77 *vs*. 70 km/h), up to expert soccer players (98 *vs*. 86 km/h) [9,15,19] and over different kick types [15]. Competition level shows to be a factor that affects *maximum* ball kicking speed [20], likely because of the influence of experience.

Therefore, the aim of the current study was: a) to determine *maximum* ball kicking speed with dominant and non-dominant leg for the two most often used kicks (instep and side-foot kick) among today’s different age categories in soccer and b) to examine differences in the ball kicking speed between first team and reserve players using specific techniques.

## Materials and methods

### Participants

One hundred and nineteen male participants that play in Croatian youth soccer leagues were recruited for participation in the present investigation. Written consent for participation in this study was obtained from the subject’s parents/guardians after being thoroughly informed about the purpose, benefits, and potential risks of this study. Consent forms were specifically approved by the “The Ethical Committee of the Faculty of Kinesiology” (Split, Croatia). This committee approved the entire study design, which was conducted according to the ethical standards of the 1964 Helsinki Declaration and its subsequent amendments.

Inclusion criteria to participate in the study were: i) participation in at least 85% of the training sessions, ii) regularly participating in the previous competitive seasons, iii) having a valid sport medical certification, and iv) being healthy (no pain or injury) and clear of any drug consumption. All players had Croatian Soccer Federation identity card signed and were fully healthy and medically examined by a local sport specialist doctor. Participants refrained from drinking caffeine-containing beverages for 24 hours and did not eat for 2 hours prior to testing in order to reduce any possible interference with the experiment.

### Design

This study is a cross-sectional investigation with the two main objectives: to determine ball kicking speed with dominant and non-dominant leg with two types of soccer kick and to examine differences of players involved in different age categories. Participants were divided according to different age groups (U-15, U-17, and U-19) and team *status* (first team, reserves). First team players were defined as the starters. Effective playing time was not taken into consideration. The current research took place in June at the end of competition season 2014/2015. Each participant completed all trials in the same time period of the testing day and under the same climate conditions (4–7 p.m., 25.6±0.8°C temperature, and 36.3±2.5% relative humidity). Participants were asked to avoid any stressful activity during testing or between training sessions.

### Procedures

Anthropometric data were measured with a portable stadiometer (SECA, Leicester, UK; for height) and an electronic scale (HD-351, Tanita, Arlington Heights, USA; for body mass) [21]. Testing protocol included a standard warm-up of 45 minutes (with 50% of theoretical maximal heart rate [220-age in yrs] as target value). Warm-up included sequences of 10 minutes of jogging with and without the ball as well as 10 minutes of dynamic stretching with a strong *focus* on leg and abdominal muscles.

During the last 15 minutes, approaching testing and for familiarization purpose [8], participants were passing and shooting with the instep kick and the side-foot kick using dominant and non-dominant leg, alternatively. Players slowly increased their kicking speed as warm-up progressed as well as kicking distance. Testing took place on the artificial grass during dry and warm weather and ball was placed on the 11-m spot. Participants were wearing their own soccer shoes and the balls used were Jabulani football (Adidas, Germany; 69.0±0.2 cm in circumference and 440±0.2 g in mass). Participants shot the ball three times with instep kick and side-foot kick using dominant and non-dominant leg, alternatively, which makes a total of 12 shots. Fastest kick *per* each type/leg was considered for further analysis. Players, lined up behind the 11-m spot, were instructed to shoot one after the other the ball as fast as they could and straight to the centre of the goal. After each player would shoot the ball, he would go to the end of the line to avoid any potential influence of fatigue. That way, every player had minimum of 3 minutes between repeated shots.

During the tests, a sport scientist was involved to better control and manage tasks. He had to give instruction: “Ready–Set–Go”, so at “Go” the player started the running kick, while another sport scientist took the measures with a pocket radar (Pocket Radar, Inc. Santa Rosa, California), with ±2 km/h accuracy, 1-m behind the goal at ball height during the kick [22].

### Statistical analysis

Basic descriptive statistics were calculated and namely as means or average score (AS), standard deviation (SD), range–*minimum* and *maximum* results (Min., Max.) for the anthropometric *status* of the participants. Systematic *bias* of kicking performance variables was determined by using 1-way analysis of variance (ANOVA) for repeated measures, with Bonferroni *post-hoc* test for eventual significant comparisons. For relative reliability intraclass correlation coefficient (ICC) with 95% confidence interval (95%CI) was calculated from ANOVA as (MSB–MSW)/MSB where MSB and MSW are mean-square variance between-individual and within-individual, respectively. ICC values less than 0.50, between 0.50 and 0.75, between 0.75 and 0.90, and greater than 0.90 are indicative of poor, moderate, good, and excellent reliability, respectively [23]. Differences in kicking speed between the first team and the reserves were determined with Student *t*-test with 95%CI for mean differences between groups. To evaluate the magnitude of differences, the Cohen’s effect size was calculated. Threshold values to interpret the effect size were <0.25 (trivial), 0.25 to 0.50 (small), 0.50 to 1.0 (moderate), and >1.0 (large) [24]. The diagnostic ability of the different ball kicking speed tests (IKDL–instep kick dominant leg, SFKDL–side foot kick dominant leg, IKNL–instep kick non-dominant leg, and SFKNL–side foot kick non-dominant leg) in capturing differences between first team players and reserves among different age categories was computed performing the receiver operating characteristics (ROC) analysis. We were aware that for instep kick *maximum* ball kicking speed is more relevant, whereas side foot kick is more suited in case of accuracy demands [8]. Nevertheless, we took into consideration side foot kick *maximum* ball kicking speed as well, because we assumed a higher ball kicking speed could make even side foot kick more effective. Area under the curve (AUC) was computed together with its 95%CI and its standard error (SE) according to the DeLong’s technique [25]. Pairwise comparisons were performed adjusting for multiple comparisons. Figures with a *P*-value of less than 0.05 were regarded as statistically significant. All statistical analyses were carried out using the commercial Statistica software version 13.0 (Dell Inc., Round Rock, TX USA).

Data for the tables in this paper are available in the online material (S1 Table).

## Results

The current study included 119 young (mean age 16.20±1.33 years) soccer players from first and second Croatian league for pioneers (U-15, *n* = 32, 26.9% of the sample), cadets (U-17; *n* = 51, 42.9%) and juniors (U-19, *n* = 36, 30.3%).

Anthropometric data are presented in Table 1. Body height, mass, and mass index values are in line with age category. The average values for height and mass for the U-15 players were just under the 75^th^ percentile (U-17 were on the 75^th^ percentile) according to American reference values [26,27]. Players were involved in soccer training programme four times a week and played one competitive match *per* week. Total weekly training/match volume for U-15, U-17, and U-19 players was 7.1±0.4 hours, 7.6±0.5 hours, 8.5±0.4 hours, respectively. During soccer season, players actively trained for 44 weeks.

**Table 1:** Anthropometric data for all participants.

Characteristic	U– 15 (*n* = 32)		U– 17 (*n* = 51)		U– 19 (*n* = 36)	
	Mean±SD	Range	Mean±SD	Range	Mean±SD	Range
**Age (years)**	14.43±0.57	13.4–15.1	16.28±0.55	15.5–17.3	17.67±0.41	16.6–18.7
**BMI**	20.33±2.24	13.9–24.72	21.92±1.79	18.21–26.37	22.08±1.59	17.74–24.74
**Height (cm)**	170.36±9.18	154.8–194.3	178.25±6.53	160–195.5	179.2±6.09	167–192.2
**Body mass (kg)**	59.45±10.57	34.7–82.6	69.69±7.01	51.8–83.7	70.95±6.86	56.6–89.5
**Soccer experience**	7.72±1.17	4–9	9.14±0.96	7–11	10.64±1.1	9–13

data presented as Mean Standard Deviation (SD) and Range for each variable, BMI–body mass index.

All 4 types of soccer kick had moderate-to-excellent ICC values (0.67–0.96), as shown in Table 2. *Maximum* trial scores were used for subsequent analyses, except for U-15 SFKNL where *post-hoc* comparison with Bonferroni correction showed significant difference among three consecutive soccer kicks (1 trial *vs*. 2 trial; *P*<0.01).

**Table 2:** Reliability of specific shooting tests.

Variable	U– 15 (*n* = 32)			U– 17 (*n* = 51)			U– 19 (*n* = 36)		
	ICC	95%CI	*F*(*P*)	ICC	95%CI	*F*(*P*)	ICC	95%CI	*F*(*P*)
IKDL	0.96	0.92 to 0.98	1.24(0.3)	0.88	0.8 to 0.93	1.39(0.26)	0.67	0.4 to 0.81	3.33(0.05)
SFKDL	0.93	0.87 to 0.96	0.24(0.79)	0.95	0.9 to 0.96	0.2(0.83)	0.77	0.6 to 0.88	0.64(0.53)
IKNL	0.91	0.85 to 0.96	1.15(0.32)	0.92	0.87 to 0.95	0.49(0.61)	0.87	0.77 to 0.93	1.33(0.27)
SFKNL	0.93	0.87 to 0.97	6.72(0.02)	0.91	0.87 to 0.95	0.26(0.78)	0.80	0.66 to 0.9	0.07(0.92)

IKDL–instep kick dominant leg; SFKDL–side foot kick dominant leg; IKNL–instep kick non-dominant leg; SFKNL–side foot kick non-dominant leg, ICC–intraclass correlation coefficient, 95%CI– 95% confidence interval, *F*–test (ANOVA), *P*–value.

Ball kicking speed among different age categories and differences between the first team players and the reserves is shown in Table 3. In U-15 age category, between first team and reserve players, results were significantly different among the three soccer kicks groups: (IKDL, *t* = -3.86, *P*<0.01; SFKDL, *t* = -3.82, *P*<0.01; IKNL, *t* = -1.87, *P* = 0.07; and SFKNL, *t* = -2.75, *P*<0.05). However, in U-17 significant differences between groups were detected in all soccer kicks (IKDL, *t* = -4.52, *P*<0.01; SFKDL, *t* = -2.41, *P*<0.05; IKNL, *t* = -4.94, *P*<0.01; and SFKNL, *t* = -2.43, *P*<0.05). Only IKDL (*t* = -2.53, *P*<0.01) could differentiate first team and reserves in U-19 age category, while in other kicks we found no significant differences (SFKDL, *t* = -0.78, *P* = 0.44; IKNL, *t* = -1.92, *P* = 0.06; and SFKNL, *t* = -0.93, *P* = 0.36).

**Table 3:** Ball kicking speed among different age categories and differences between the first team players and the reserves.

	Soccer kick type	Mean±SD (km/h)		*t*	ES	95%CI
		First team Mean±SD	Reserves Mean±SD			
U– 15 (*n* = 32)	IKDL	100.27±4.76	90.59±8.61	**-3.86**^***b***^	1.58 (very large)	-4.56 to -14.8
	SFKDL	90.93±6.54	78.0±11.59	**-3.82**^***b***^	1.35 (very large)	-6 to -19.9
	IKNL	87.73±6.49	82.71±8.42	-1.87	0.71(medium)	0.45 to -10.5
	SFKNL	80.27±6.69	71.0±11.42	**-2.75**^***a***^	1 (large)	-2.38 to -16.1
U– 17 (*n* = 51)	IKDL	107.43±4.65	100.86±5.56	**-4.52**^***b***^	1.55 (very large)	-3.6 to -9.5
	SFKDL	96.39±7.51	90.93±8.46	**-2.41**^***a***^	0.8 (large)	-0.9 to -10
	IKNL	96.83±5.59	86.46±8.68	**-4.94**^***b***^	1.5 (very large)	-6.2 to –14.6
	SFKNL	87.87±7.58	82.82±7.2	**-2.43**^***a***^	0.7 (medium)	-0.9 to -9.2
U-19 (*n* = 36)	IKDL	111.3±4.35	106.94±6.15	**-2.49**^***a***^	1 (large)	-0.8 to -7.9
	SFKDL	98.9±6.43	97.19±6.77	-0.78	0.2 (small)	2.78 to -6.2
	IKNL	99±6.5	94.69±6.94	-1.92	0.8 (large)	0.25 to -8.9
	SFKNL	90.2±6.07	88.13±7.38	-0.93	0.3 (small)	2.48 to -6.6

data presented as Mean ± Standard Deviation (SD), *t*-Student test of differences, ES- Cohen’s *d* effect size,

^a^ *P*<0.05,

^b^ *P*<0.01, 95%CI– 95% confidence interval, IKDL–instep kick dominant leg; SFKDL–side foot kick dominant leg; IKNL–instep kick non dominant leg; SFKNL–side foot kick non dominant leg.

When comparing all soccer kicks, participants achieved highest speed with IKDL (U-15 first team 100.27±4.76 km/h *vs*. reserves 90.59±8.61 km/h; U-17 first team 107.43±4.65 km/h *vs*. reserves 100.86±5.56 km/h; and U-19 first team 111.3±4.35 km/h *vs*. reserves 106.94±6.15 km/h) while, as expected, the lowest speed was with SFKNL (U-15 first team 80.27±6.69 km/h *vs*. reserves 71±11.42 km/h; U-17 first team 87.87±7.58 km/h *vs*. reserves 82.82±7.2 km/h; and U-19 first team 90.2±6.07 km/h *vs*. reserves 88.13±7.38 km/h).

Concerning the ROC analysis, for U-15, AUC ranged from 0.673 (IKNL) to 0.837 (SFKDL), whereas, for U-17, AUC ranged from 0.680 (SFKDL) to 0.819 (IDKL), and, for U-19, AUC ranged from 0.584 to 0.780. Further details are reported in Table 4.

**Table 4:** Pairwise comparisons of AUC curves.

	Variable	AUC	SE	95% CI
U– 15 (*n* = 32)	IDKL	0.80	0.081	0.63 to 0.92
	IKNL	0.67	0.097	0.49 to 0.83
	SFKDL	0.84	0.072	0.66 to 0.94
	SFKNL	0.76	0.088	0.58 to 0.89
U– 17 (*n* = 51)	IDKL	0.82	0.061	0.69 to 0.91
	IKNL	0.87	0.051	0.75 to 0.95
	SFKDL	0.68	0.077	0.54 to 0.80
	SFKNL	0.69	0.076	0.54 to 0.81
U-19 (*n* = 36)	IDKL	0.78	0.090	0.61 to 0.90
	IKNL	0.70	0.092	0.53 to 0.84
	SFKDL	0.58	0.098	0.41 to 0.75
	SFKNL	0.61	0.101	0.43 to 0.77

AUC–area under the curve; 95%CI– 95% confidence interval; SE–standard error; IKDL–instep kick dominant leg; SFKDL–side foot kick dominant leg; IKNL–instep kick non-dominant leg; SFKNL–side foot kick non-dominant leg.

**Fig 1:**
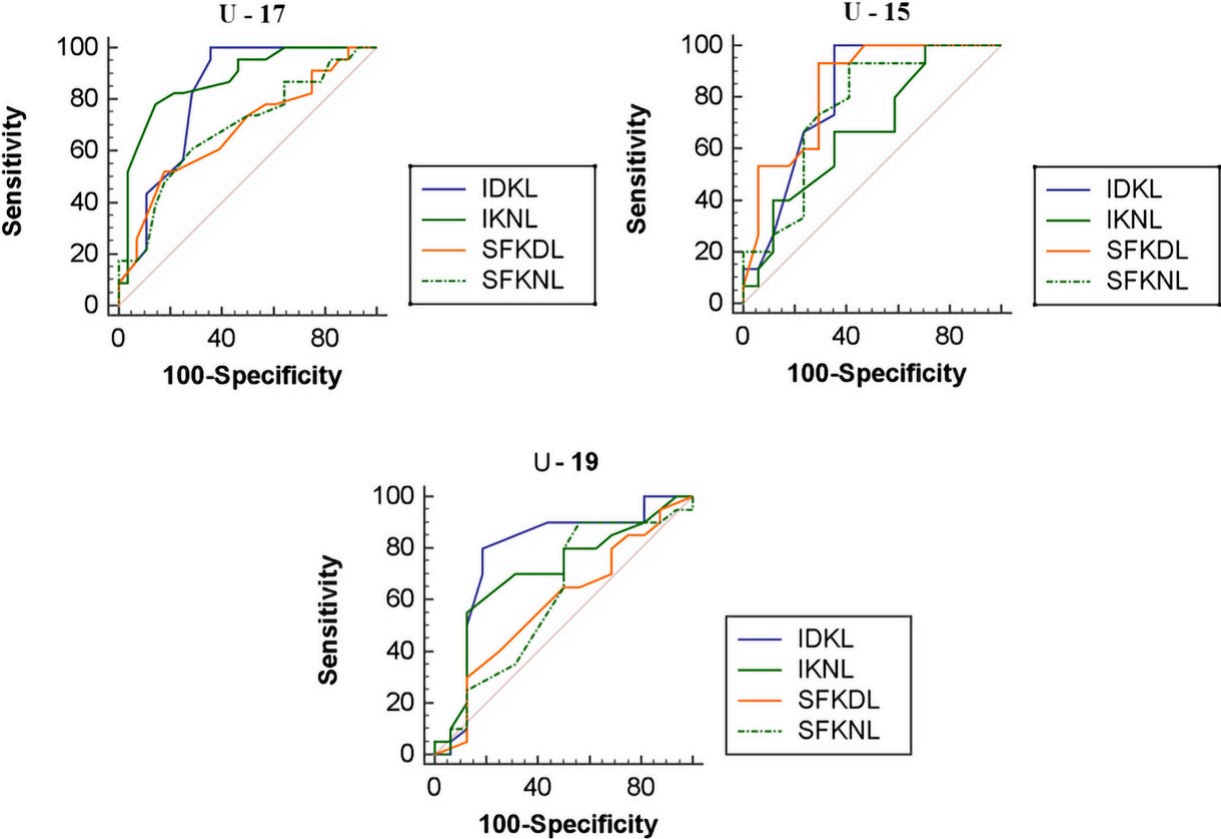
ROC analysis broken down for U-15, U-17 and U-19 players. IKDL–instep kick dominant leg; SFKDL–side foot kick dominant leg; IKNL–instep kick non-dominant leg; SFKNL–side foot kick non-dominant leg.

At the pairwise comparison of ROC curves (Fig 1), for U-15, IDKL did not differ from IKNL (Δ = 0.131 [95%CI -0.0861–0.349], SE = 0.111, *z* = 1.184, *P* = 0.2364), from SFKDL (Δ = 0.0333 [95%CI -0.0862–0.153], SE = 0.0610, *z* = 0.546, *P* = 0.5848), and from SFKNL (Δ = 0.0451 [95%CI -0.0952–0.185], SE = 0.0716, *z* = 0.630, *P* = 0.5287). Similarly, IKNL did not differ from SFKDL (Δ = 0.165 [95%CI -0.0466–0.376], SE = 0.108, *z* = 1.528, *P* = 0.1265) and from SFKNL (Δ = 0.0863 [95%CI -0.125–0.298], SE = 0.108, *z* = 0.799, *P* = 0.4242). Finally, SFKDL did not differ from SFKNL (Δ = 0.0784 [95%CI -0.0684–0.225], SE = 0.0749, *z* = 1.047, *P* = 0.2950).

For U-17, IDKL did not differ from IKNL (Δ = 0.0536 [95%CI -0.0838–0.191], SE = 0.0701, *z* = 0.764, *P* = 0.4448) and from SFKNL (Δ = 0.131 [95%CI -0.0365–0.299]], SE = 0.0855, *z* = 1.534, *P* = 0.1251), but differed from SFKDL (Δ = 0.139 [95%CI 0.00826–0.270], SE = 0.0667, *z* = 2.084, *P* = 0.0372). IKNL differed both from SFKDL (Δ = 0.193 [95%CI 0.0351–0.350], SE = 0.0804, *z* = 2.396, *P* = 0.0166) and from SFKNL (Δ = 0.185 [95%CI 0.0437–0.326], SE = 0.0720, *z* = 2.566, *P* = 0.0103). Finally, SFKDL did not differ from SFKNL (Δ = 0.00776 [95%CI -0.132–0.148], SE = 0.0713, *z* = 0.109, *P* = 0.9133).

For U-19, IDKL differed only from SFKDL (Δ = 0.195 [95%CI 0.0392–0.351], SE = 0.0796, *z* = 2.453, *P* = 0.0142), but not from IKNL (Δ = 0.0766 [95%CI -0.0805–0.234], SE = 0.0801, *z* = 0.956, *P* = 0.3392) and from SFKNL (Δ = 0.172 [95%CI -0.0265–0.370], SE = 0.101, *z* = 1.698, *P* = 0.0895). IKNL did not differ from SFKDL (Δ = 0.119 [95%CI -0.0927–0.330], SE = 0.108, *z* = 1.101, *P* = 0.2710) and from SFKNL (Δ = 0.0953 [95%CI -0.0734–0.264], SE = 0.0861, *z* = 1.107, *P* = 0.2683). Finally, SFKDL did not differ from SFKNL (Δ = 0.0234 [95%CI -0.155–0.202], SE = 0.0912, *z* = 0.257, *P* = 0.7972).

## Discussion

The main objectives of this study were to determine ball kicking speed with dominant and non-dominant leg for two commonly used soccer kicks in different age categories and to determine differences in ball kicking speed between first and second team players. In this study, more successful players (first team) achieved higher ball speeds when compared to reserves. Looking at the results of statistical significance, differences were greater at U-15 and U-17 players in relation to the U-19 category. The magnitude of the differences between the first team and reserve players were very large-to-large (U-15), very large-to-medium (U-17), and large-to-small (U-19). Differences in ball kicking speed between the first team players and the reserves seem to diminish over age.

Interestingly, actual findings were different from previous ones in numerous studies, in which it were measured ball kicking performance without taking into consideration age groups [16,28–30]. Differently, Rodríguez-Lorenzo et al.’s study–taking into account fewer age groups–provided kicking speeds similar to current study’s ones [31]. Also, majority of studies measured ball speed of the instep kick [28,29,32–34]. To the best of our knowledge, there is only one study that evaluates kicking speed among soccer players at different competition levels [35], but included *senior* soccer players and did not find differences between division 1, division 2, and *amateur* players.

Results of AUC for instep kick dominant leg for all categories of age were higher than instep kick non-dominant leg and side foot kick dominant and non-dominant leg and confirm, by using ROC analysis, existing literature reporting that *maximum* speed can mainly be reached when using dominant leg and kicking with the laces of the foot [2,9,10]. This finding is applicable for all age categories. In the other hand, side foot kick dominant and non-dominant leg AUC results were found to be lower in all groups and especially in aged ones, confirming that there is an effect of age of the muscle contraction speed with advancing age [36]. Such findings confirm to be very interesting since they hint that even an eventual detection of a slight reduction in speed of kicking in a more aged group (e.g., 17 *vs*. 15 years of age) should be taken into consideration.

By looking separately at different age categories for U-15 players of the first team and reserves, the ball kicking speeds achieved with IKDL are better than those reported by Marques et al. (100.3 km/h and 87.7 km/h *vs*. 84.6 km/h [37]). For the U-17 players there are many variations in the results of IKDL. Juárez et al. [28] reported a speed of 108.22 km/h, which is similar to that obtained by the U-17 players of the first team in this study but better than the result achieved by the reserves. Nunome et al. [38] with the elite U-17 players got a kicking speed of 115.6 km/h, while lower scores were obtained in the studies by Tomáš et al. [29] and by García-Pinillos et al. [34], 102.89 km/h and 84.85 km/h, respectively. Soccer kicking is an extremely important part of the game and in youth academies is often taken as one of the key determinants for assessing the quality and selecting talents. Players of the first team kicked faster shots than the reserves for all age categories and the differences were statistically significant and much higher among U-15 and U-17 players. It may be noted that the differences in the kicking speed between the first team players and reserves tended to decrease over age. These results may be an indicator of the influence of some other variables underlying the performance of players. Potential reason for this could be variation in time of growth spurt and maturation among young boys. Players who biologically develop earlier are often selected in more successful teams and in first teams [39–48]. It is confirmed that, because of the differences in biological *status*, more mature players have more muscle mass and are able to generate more power and higher acceleration at the ends of the limbs and achieve faster kicks. Such a finding suggests that assessing kicking speed in older age categories would not make particular sense because performed after coaches and trainers’ relative age effect- and biological *status*-biased recruitment that makes older players more homogenous in terms of kicking speed [48]. Differently, assessing kicking speed in younger age categories could make some sense, because it could better highlight genuine differences in young players not yet affected by coaches and trainers’ future cut (and therefore in a sample more heterogeneous in terms of skills) [31]. Within U-19 category, only with instep kick with dominant leg there was statistically significant difference between first and second team players. Such results could be an indication of how the differences decrease as you approach the *senior*/professional *status* of players. As age increases, there is also much more selection of the players so there are also less differences between first team and reserve players. Soccer kicking speed could potentially be one of the performance indicators and selection tool in youth soccer, particularly at younger ages, but needs to be evaluated taking several other aspects (*in primis*, biological *status*) into consideration.

A limitation of this study was that we used a too long-lasting warm-up (45 min) for a kicking protocol. A shorter a 10-minute warm-up *plus* 5-minute active stretching would have been more appropriate for our protocol [2]. Another limitation was that we did not check for fatigue evidence (e.g., by continuously monitoring players’ heart rate) over protocol administration. Another way for controlling for fatigue advent could have been to allow each player deciding his own pace over protocol [20].

## Conclusions

For young players of different age categories, statistically significant differences in ball kicking speed for instep and side-foot kicks with dominant and non-dominant leg between the first team and the reserves were found. In this research, the fastest kick was instep kick with dominant leg whereas the slowest was side-foot kick with non-dominant leg. Also, significant differences between the first team and reserves could be detected in groups U-15 and U-17 when compared with the older group U-19. Previously research seems to suggest the influence of certain biological factors such as growth spurt and maturation on the selection for the first team. Soccer kicking can be a quality indicator for assessing the soccer skill performance of players. By assessing soccer kicking performance, relevant results can be obtained in a fast, easy, and efficient way and can be utilized in the selection process of young talents in soccer.

## Supporting information

S1 Table Data. Data for the tables.
(XLSX)
